# A 12-week randomized, double-blind, placebo-controlled multicenter study of choline-stabilized orthosilicic acid in patients with symptomatic knee osteoarthritis

**DOI:** 10.1186/s12891-016-1370-7

**Published:** 2017-01-05

**Authors:** Piet Geusens, Karel Pavelka, Jozef Rovensky, Johan Vanhoof, Nathalie Demeester, Mario Calomme, Dirk Vanden Berghe

**Affiliations:** 1 Biomedical Research Institute (BIOMED), Hasselt University, Diepenbeek, Belgium; 2 Rheumatology, Maastricht UMC, Maastricht, The Netherlands; 3 ReumaClinic, Bretheistraat 149, Genk, 3600 Belgium; 4 Institute of Rheumatology, Prague, Czech Republic; 5 Nábrežie I. Krasku 4782/4, Piešťany, Slovakia; 6 Research & Development, Bio Minerals NV, Destelbergen, Belgium; 7 Department of Pharmaceutical Sciences, University of Antwerp, Antwerp-Wilrijk, Belgium

**Keywords:** Osteoarthritis, Knee, Choline-stabilized orthosilicic acid, WOMAC, Cartilage degradation marker, Silicon, Choline

## Abstract

**Background:**

The aim of this study was to assess the efficacy of choline-stabilized orthosilicic acid (ch-OSA) in patients with symptomatic knee osteoarthritis (OA).

**Methods:**

In a multicenter, double-blind, placebo-controlled study, 211 patients with knee OA (Kellgren and Lawrence grade II or III) and moderate to moderately severe pain were randomly allocated to ch-OSA or placebo for 12 weeks. The primary outcome was the change in the WOMAC pain subscale from baseline to week 12. Secondary outcomes were changes from baseline to week 12 in WOMAC total, WOMAC stiffness, WOMAC physical function, Subject Global Assessment and levels of cartilage degradation biomarkers C-terminal telopeptide of collagen type II (CTX-II) and cartilage oligomeric matrix protein (COMP). Pre-specified subgroup analyses included the effect of gender.

**Results:**

A total of 166 (120 women, 46 men) patients were included in the analysis (87 and 79 in the ch-OSA and placebo group, respectively). In the total study population, no differences were observed between the two treatment groups for the different outcomes but significant treatment x gender interactions were found. In men taking ch-OSA, a significant improvement in WOMAC total, WOMAC stiffness and WOMAC physical function as well as a lower increase in biomarker levels of cartilage degradation was observed, but not in women. The change in WOMAC pain showed a similar positive trend in men taking ch-OSA.

**Conclusion:**

After 12 weeks of treatment, no effect was found of ch-OSA in the total study population on clinical parameters and biomarkers, but a gender interaction was observed. In men, ch-OSA was found effective in reducing symptoms of knee OA, which was associated with a slight but significant reduction of biomarkers that are related to cartilage degradation.

**Trial registration:**

The study was registered retrospectively: ISRCTN88583133. Registration date: 2015-10-07.

**Electronic supplementary material:**

The online version of this article (doi:10.1186/s12891-016-1370-7) contains supplementary material, which is available to authorized users.

## Background

Osteoarthritis (OA) is one of the leading causes of functional disability and compromised quality of life in the elderly worldwide [1]. It is a chronic and slowly progressive disease, with the knee being the most affected weight-bearing joint [2, 3]. OA is a complex disease of joints, characterised by degradation of articular cartilage, changes of the subchondral bone and inflammation, leading to pain and stiffness in the joint [4, 5]. At present, there are no safe OA therapeutic strategies approved that result in a concurrent structural modification and symptom improvement [6, 7]. The treatment of OA is therefore, besides exercise and physiotherapy, still mainly based on analgesics, non-steroidal anti-inflammatory drugs (NSAIDs) and surgical procedures [8].

Several risk factors for the development of knee OA have been identified, including age, obesity, injury and genetic profiles. Sex differences in knee OA incidence and prevalence have been reported as well, with females generally at higher risk [7, 9]. Moreover, the meta-analysis performed by Srikanth et al. demonstrated that women tend to have more severe knee OA, particularly after menopausal age [9].

Given the limitations of plain radiography and the high charges for magnetic resonance imaging (MRI), biochemical markers have gained considerable interest over the past 15 years [10, 11]. However, in contrast to the well-established biochemical markers for bone diseases, the value of biomarkers in OA still needs further elucidation [12]. To this respect, Bauer et al. proposed the BIPED – burden of disease, investigative, prognostic, efficacy of intervention and diagnostic – categories, providing a classification system for biochemical markers [13]. Among numerous candidates, C-terminal telopeptide of collagen type II (CTX-II) and cartilage oligomeric matrix protein (COMP), which are two markers of cartilage degradation, have been reported to be the best performing biochemical markers across all BIPED categories [11].

Choline-stabilized orthosilicic acid (ch-OSA) is a specific complex of orthosilicic acid and choline that has been previously demonstrated to stimulate collagen synthesis which positively affects bone turnover [14] as well as surface and mechanical properties of the skin [15]. The choline component of ch-OSA was suggested to contribute to this effect [14], as it lowers plasma homocysteine levels [16]. More specifically, hyperhomocysteinemia has been shown to interfere with collagen cross-linking, leading to connective tissue pathology [17]. To this respect, it may be hypothesized that ch-OSA acts on both cartilage and subchondral bone, consequently rendering it a candidate for the treatment of OA. The aim of this study was to evaluate the symptomatic effects of the oral intake of ch-OSA on knee OA over a 12-week period. Additionally, CTX-II and COMP were analysed to assess the influence of ch-OSA on cartilage degradation.

## Methods

A 12-week, international, multicenter, double-blind, randomized, parallel-group, placebo-controlled single-joint study in outpatients with symptomatic knee OA was performed. Participants were recruited either directly from the investigation centres (situated in Belgium (Reuma*Clinic*, Genk and Diagnosecentrum, Lommel; both private hospitals in co-operation with Biomedical Research Institute, Hasselt University, Hasselt, Belgium and Rheumatology, Maastricht UMC, Maastricht, The Netherlands), Slovakia (National Institute of Rheumatic Diseases, Piestany; public hospital) and Czech Republic (Institute of Rheumatology, Prague; public hospital) or following pre-screening by general practitioners (Belgium).

### Patients

Potential participants were initially assessed during a screening visit. The inclusion criteria defined eligible patients as men and women between 50 and 75 years of age with a diagnosis of primary knee OA according to the American College of Rheumatology criteria [18] for at least 12 weeks prior to randomization i.e. knee pain plus at least three of the following characteristics: > 50 years, < 30 min of morning stiffness, crepitus on active motion, bony tenderness, bony enlargement, no palpable warmth of the synovium. Additional inclusion criteria comprised a radiographic confirmed Kellgren and Lawrence grade II or III (mild to moderate osteophytes and joint space narrowing) in the previous 6 months [19]; a knee OA pain intensity score, assessed by the question “How would you describe your maximum OA knee pain when not taking analgesic medications in the 24 h prior to this visit”, of “moderate (2)” or “moderately severe (3)” on a 5-point Likert Scale; a Western Ontario and McMaster University OA Index (WOMAC) physical function subscale score > 0 on a 100 mm horizontal visual analogue scale (VAS) [20]. In case both knees were affected, the knee with the highest pain score was selected as the target knee. In order to avoid interference with the subject’s pain perception, the use of paracetamol within a 48 h period prior to the screening visit was not allowed.

The major exclusion criteria were the following: secondary OA of the target knee; morning stiffness of ≥ 30 min; a swollen or warm joint suspected to be secondary to gout, pseudo gout or sepsis; significant injury in the target joint within 6 months before the start of the trial; arthroplasty and joint surgery of the target knee within 2 years prior to the start of the study. Additionally, specified previous medications led to exclusion from the trial.

Following a wash-out period during which the use of pain medication and OA-related treatments were not allowed, eligible subjects were scheduled for a baseline visit. The duration of the wash-out period was at least five drug half-lives of the corresponding drug, or as described in the exclusion criteria.

### Design, randomisation and blinding

At the baseline visit, patients were randomly assigned to take a capsule of either ch-OSA (520 mg ch-OSA beadlets containing 5 mg of silicon and 100 mg of choline; Bio Minerals N.V., Belgium) or placebo (520 mg microcrystalline cellulose beadlets; Pharmatrans Sanaq AG, Switzerland) twice daily by oral route for 12 weeks. The treatment allocation occurred sequentially in a 1:1 ratio using a randomization list, which was generated by an independent statistician in R (software version 2.10.1 for Windows; The R Foundation for Statistical Computing, Vienna, Austria). More specifically, block randomization was stratified by site, applying randomly selected block sizes of 2 or 4. The individual code was kept in a sealed envelope by the investigator to be opened only in case of medical emergency.

Patients were assessed by the investigator at baseline and at 2, 6 and 12 weeks after randomization. At the first three visits, the study dietary supplement was delivered in bottles labelled with the subject’s randomization number (according to the allocation sequence) and the instructions to take one capsule in the morning and another in the evening with a glass of water or juice. Blinding among subjects, investigators and monitors was maintained by providing identical packaging, appearance, taste and odour for ch-OSA and placebo capsules, respectively. Treatment compliance was verified at subsequent visits by counting the number of unused doses. In this respect, the limit for an acceptable compliance was set at 75%.

Pain medication and OA-related treatments were not allowed throughout the trial. However, subjects were allowed to take up to 2 g of paracetamol per day as rescue medication in case of intolerable pain, except during a 48 h period before each evaluation.

### Assessments and outcome measures

At each visit, allocated patients were requested to complete a 100 mm horizontal VAS WOMAC questionnaire based on symptoms in the target knee in the preceding 48 h. This 24-item questionnaire resulted in a total WOMAC score and three WOMAC subscores, based on the following subscales: pain (5 questions), stiffness (2 questions) and physical function (17 questions). For each WOMAC score, a 0–100 mm range was generated by averaging across the respective items, with lower scores indicating better outcomes. The Subject Global Assessment was evaluated on a 100 mm horizontal VAS as well (0–100 mm range), by asking the following question: “Considering all the ways your knee OA affects you, how are you doing today?”. Finally, fasting serum and second void urine were collected at the baseline and final visit to assess biochemical markers of cartilage degradation (urine, serum), baseline serum estradiol and serum silicon concentration. For the collection of serum, silicon free polypropylene syringes (Sarstedt, Germany) and needles (Microlance, Becton Dickinson, Spain) were used as published elsewhere [15]. Aliquots of both samples were stored at −20 °C until analysis. More specifically, urinary CTX-II concentrations were determined using the Urine CartiLaps EIA from IDS (Boldon, UK). These concentrations were corrected for urinary creatinine concentrations, which were determined with a creatinine assay (R&D Systems, Abingdon, UK). Serum COMP levels were measured using AnaMar Medical’s (Lund, Sweden) COMP ELISA. Serum Estradiol levels were measured with Estradiol III electrochemiluminescense immunoassay from Roche (Mannheim, Germany). All analyses were performed according to the manufacturers’ protocols. Serum silicon concentration was measured using electrothermal atomic absorption spectrometry with inverse longitudinal Zeeman background correction (AAnalyst 800, Perkin Elmer, Bodenseewerk, Germany) and the analytical features (temperature program, matrix modifier, sample preparation method) were published elsewhere [15].

The primary outcome measure was the change in the WOMAC pain subscale from baseline to week 12. Secondary outcome measures included the changes from baseline to 12 weeks in the total WOMAC score, the WOMAC stiffness subscale, the WOMAC physical function subscale, the Subject Global Assessment and CTX-II and COMP levels.

### Statistical analysis

The sample size calculation estimated that a group size of 100 subjects was necessary to ensure at least 80% power to detect differences of 16% between the ch-OSA and placebo group with respect to pain and physical function (quantified according to the WOMAC pain and function subscale, assuming a relative standard deviation of 40% (based on data of 13 studies; Additional file 2: Table S2)), considering a dropout rate of 10% and a 0.05 two-sided significance level.

Statistical analyses were performed using the per-protocol population, defined as all randomized patients meeting the inclusion criteria, who completed the trial and who did not have major protocol violations. Results are presented as mean ± standard deviation (SD).

For WOMAC total, WOMAC subscales and Subject Global Assessment scores, a repeated measures (RM) univariate analysis of covariance (ANCOVA) model was applied in which the baseline value was treated as a covariate while treatment and gender were treated as fixed factors. Between and within treatment differences were assessed and the models also included interaction terms between treatment and gender. Differences in serum silicon and biomarker values (baseline versus final visit) were analysed between and within treatments using RM analysis of variance (ANOVA), albeit after removal of outliers. These outliers were identified using the median absolute deviation and a critical value of 4.5 [21]. In case of a significant interaction between time and treatment, post hoc t-tests with Bonferroni correction were performed.

Additionally, as specified in the study protocol, subgroup analyses by gender were performed for all outcome measures. Due to the smaller group sizes, non-parametric tests were used. More specifically, differences between treatment groups were analysed with Mann-Whitney U, whereas differences within treatment groups were analysed using Friedman’s test with post hoc Bonferroni-corrected Wilcoxon signed-ranks. Correlation of serum silicon with clinical parameters was analysed in subgroups with the Spearman rank correlation test.

Significance levels were set at *P* < 0.05 (two-sided), except for interaction tests (*P* < 0.10) [22]. All statistical analyses were performed using SPSS software (version 21.0 for Windows, IBM Corp., Armonk, NY, USA).

## Results

### Patients

**Fig. 1:**
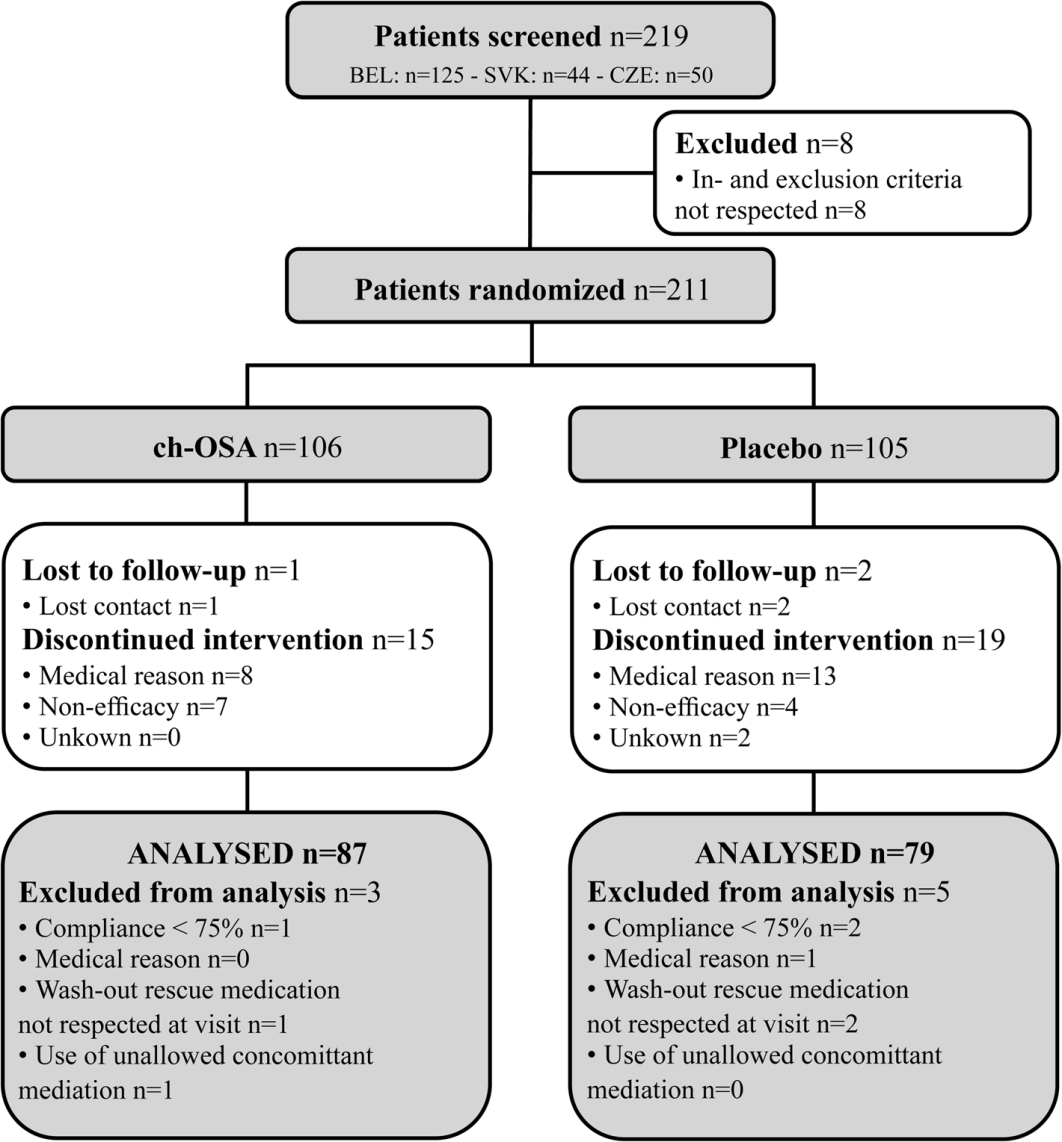
Flow diagram of study enrolment, allocation, follow-up and analysis

Between June 2010 and April 2012, a total of 211 eligible subjects were randomized and allocated to receive ch-OSA (*n* = 106) or a placebo (*n* = 105). As demonstrated in Fig. 1, 166 patients were ultimately included in the per-protocol analysis. Demographic variables, baseline disease characteristics and baseline outcome measures were similar in both groups (Table 1). Mean age of the randomized patients was 61.9 ± 7.1 years with a majority of postmenopausal women. The mean baseline serum level of estradiol was significantly (*P* < 0.0005) lower in women compared to men (Table 1). Baseline CTX-II levels showed a higher trend (*P* = 0.1) in women compared to men (Table 1).

**Table 1:** Baseline characteristics of the study population by treatment group and gender

Characteristic	Placebo (*n* = 79)	ch-OSA (*n* = 87)	Male (*n* = 46)	Female (*n* = 120)
Age (years)	62.2 ± 7.7	61.7 ± 6.5	61.0 ± 7.2	62.3 ± 7.0
Women	55 (69.6)	65 (74.7)	0	120 (100)
Body mass index (kg/m^2^)	29.5 ± 5.2	29.5 ± 5.4	29.1 ± 4.7	29.6 ± 5.6
Menopausal Status	1 pre (1.5)	1 pre (1.8)	-	2 pre (1.7)
	0 peri (1.5)	1 peri		1 peri (0.8)
	54 post (98.2)	63 post (96.9)		117 post (97.5)
Kellgren and Lawrence grade II	48 (60.8)	59 (67.8)	26 (56.5)	81 (67.5)
Kellgren and Lawrence grade III	31 (39.2)	28 (32.2)	20 (43.5)	39 (32.5)
Likert pain score 2	38 (48.1)	32 (36.8)	19 (41.3)	51 (42.5)
Likert pain score 3	41 (51.9)	55 (63.2)	27 (58.7)	69 (57.5)
WOMAC Total (/100 mm)	43.1 ± 20.3	40.9 ± 19.4	42.2 ± 21.5	41.9 ± 19.2
WOMAC Pain (/100 mm)	41.2 ± 20.3	38.2 ± 19.8	41.9 ± 20.9	38.7 ± 19.7
WOMAC Stiffness (/100 mm)	45.3 ± 24.6	44.0 ± 23.5	44.9 ± 23.4	44.5 ± 24.3
WOMAC Function (/100 mm)	43.4 ± 21.2	41.4 ± 19.8	42.0 ± 22.5	42.5 ± 19.7
Subject Global Assessment (/100 mm)	49.4 ± 21.8	50.1 ± 18.2	51.3 ± 22.4	49.2 ± 19.0
CTX-II/Creatinine (ng/mmol)	413.1 ± 189.6	430.4 ± 212.7	378.5 ± 162.8	441.0 ± 213.9
COMP (U/L)	10.8 ± 2.8	10.7 ± 2.8	11.0 ± 2.8	10.6 ± 2.8
Estradiol (ng/L)	-	-	35.5 ± 31.0	22.0 ± 36.2 ^a^

Data expressed as mean ± SD or n (%);-: not applicable; ^a^ significant difference between groups (men versus women); Number of patients without outliers for CTX-II: Placebo (*n* = 66), ch-OSA (*n* = 68); Number of patients without outliers for COMP: Placebo (*n* = 74), ch-OSA (*n* = 80)

### Compliance and safety

Compliance throughout the study was excellent as 98% of the patients reached the minimum compliance of 75%. Overall, the mean compliance was 106 ± 12% in the per-protocol population and > 100% both in men and women. No adverse events related to the treatment were reported in either treatment group.

### Efficacy

The primary and secondary outcome measures are shown in Table 2, along with the absolute values of the assessed clinical outcome measures at the different visits. After 12 weeks of treatment, the change from baseline in silicon serum levels was significantly higher in the ch-OSA group compared to the placebo group (Table 2). The statistical analyses demonstrated no significant differences between the two treatment groups with respect to the primary and secondary outcome measures. Nevertheless, significant treatment x gender interactions were found for all clinical outcomes (ANCOVA).

**Table 2:** Overview of the primary and secondary outcome measures and the absolute clinical outcome scores at baseline, week 2, week 6 and week 12 in the placebo and choline-stabilized orthosilicic acid (ch-OSA) group

	Placebo (*n* = 79)					ch-OSA (*n* = 87)					Treatment difference at 12 weeks (95% CI)	*P* value	*P* value treatment x gender interaction
	Baseline (mean ± SD)	Week 2 (mean ± SD)	Week 6 (mean ± SD)	Week 12 (mean ± SD)	Change week 12 − baseline (mean ± SD)	Baseline (mean ± SD)	Week 2 (mean ± SD)	Week 6 (mean ± SD)	Week 12 (mean ± SD)	Change week 12 − baseline (mean ± SD)			
WOMAC total score (/100 mm)	43.1 ± 20.3	39.1 ± 21.3	32.7 ± 21.2	32.3 ± 23.9	−10.8 ± 20.5	40.9 ± 19.4	37.3 ± 20.9	29.5 ± 21.5	29.3 ± 22.9	−11.6 ± 15.8	0.8 (−4.8 to 6.4)	0.78	0.06
WOMAC pain score (/100 mm)	41.2 ± 20.3	36.6 ± 21.1	30.3 ± 21.1	29.7 ± 23.9	−11.5 ± 22.1	38.2 ± 19.8	34.7 ± 21.4	27.7 ± 21.8	26.1 ± 22.2	−12.1 ± 17.5	0.6 (−5.5 to 6.7)	0.85	0.07
WOMAC stiffness score (/100 mm)	45.3 ± 24.6	39.7 ± 24.3	32.7 ± 23.4	34.0 ± 25.0	−11.3 ± 24.2	44.0 ± 23.5	38.8 ± 23.1	30.5 ± 22.8	30.0 ± 24.9	−14.0 ± 19.5	2.7 (−4.0 to 9.4)	0.43	0.04
WOMAC function score (/100 mm)	43.4 ± 21.2	39.8 ± 21.8	33.4 ± 21.8	32.9 ± 24.3	−10.6 ± 20.8	41.4 ± 19.8	37.9 ± 21.2	29.9 ± 21.8	30.2 ± 23.6	−11.2 ± 16.5	0.6 (−5.1 to 6.3)	0.84	0.09
Subject Global Assessment (/100 mm)	49.4 ± 21.8	39.0 ± 19.9	33.4 ± 20.7	32.5 ± 22.7	−16.9 ± 24.3	50.1 ± 18.2	41.2 ± 22.4	33.5 ± 24.4	32.1 ± 24.5	−18.0 ± 22.7	1.2 (−6.0 to 8.4)	0.75	0.05
CTX-II/Creatinine (ng/mmol)	413.1 ± 189.6	-	-	528.0^a^ ± 261.8	114.9 ± 174.7	430.4 ± 212.7	-	-	533.6^a^ ± 263.2	103.2 ± 178.6	11.7 (−48.7 to 72.1)	0.70	-
COMP (U/L)	10.8 ± 2.8	-	-	11.3 ± 3.4	0.6 ± 2.9	10.7 ± 2.8	-	-	11.1 ± 2.8	0.4 ± 2.6	0.1 (−0.7 to 1.0)	0.75	-
Silicon (μg/L)	50.9 ± 33.6	-	-	65.4^a^ ± 42.2	14.6 ± 40.9	59.9 ± 36.1	-	-	115.8 ^a, b^ ± 56.0	55.8 ± 52.1	41.2 (25.8 to 56.8)	0.0005	-

^a^significant difference from baseline within the group; ^b^significant difference between groups (placebo versus ch-OSA); -: not applicable; Number of patients without outliers for CTX-II: placebo (*n* = 66), ch-OSA (*n* = 68); Number of patients without outliers for COMP: placebo (*n* = 74), ch-OSA (*n* = 80); Number of patients without outliers for Silicon: placebo (*n* = 69), ch-OSA (*n* = 75)

**Fig. 2:**
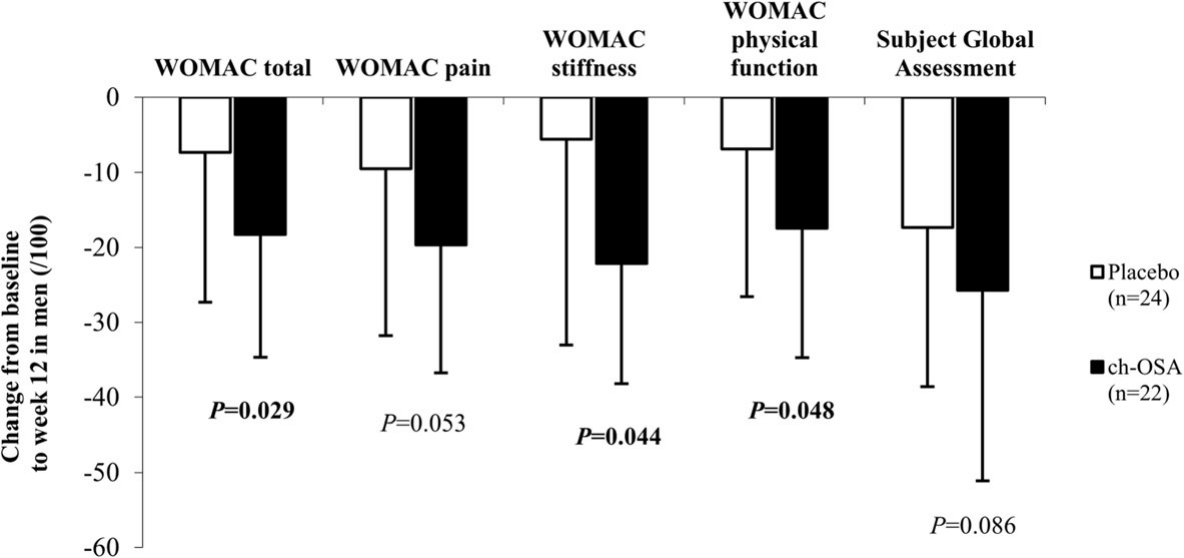
Mean change (± SD) from baseline to week 12 in WOMAC total, WOMAC pain, WOMAC stiffness, WOMAC physical function and Subject Global Assessment in men. *P* values refer to differences between placebo and choline-stabilized orthosilicic acid (ch-OSA)

**Fig. 3:**
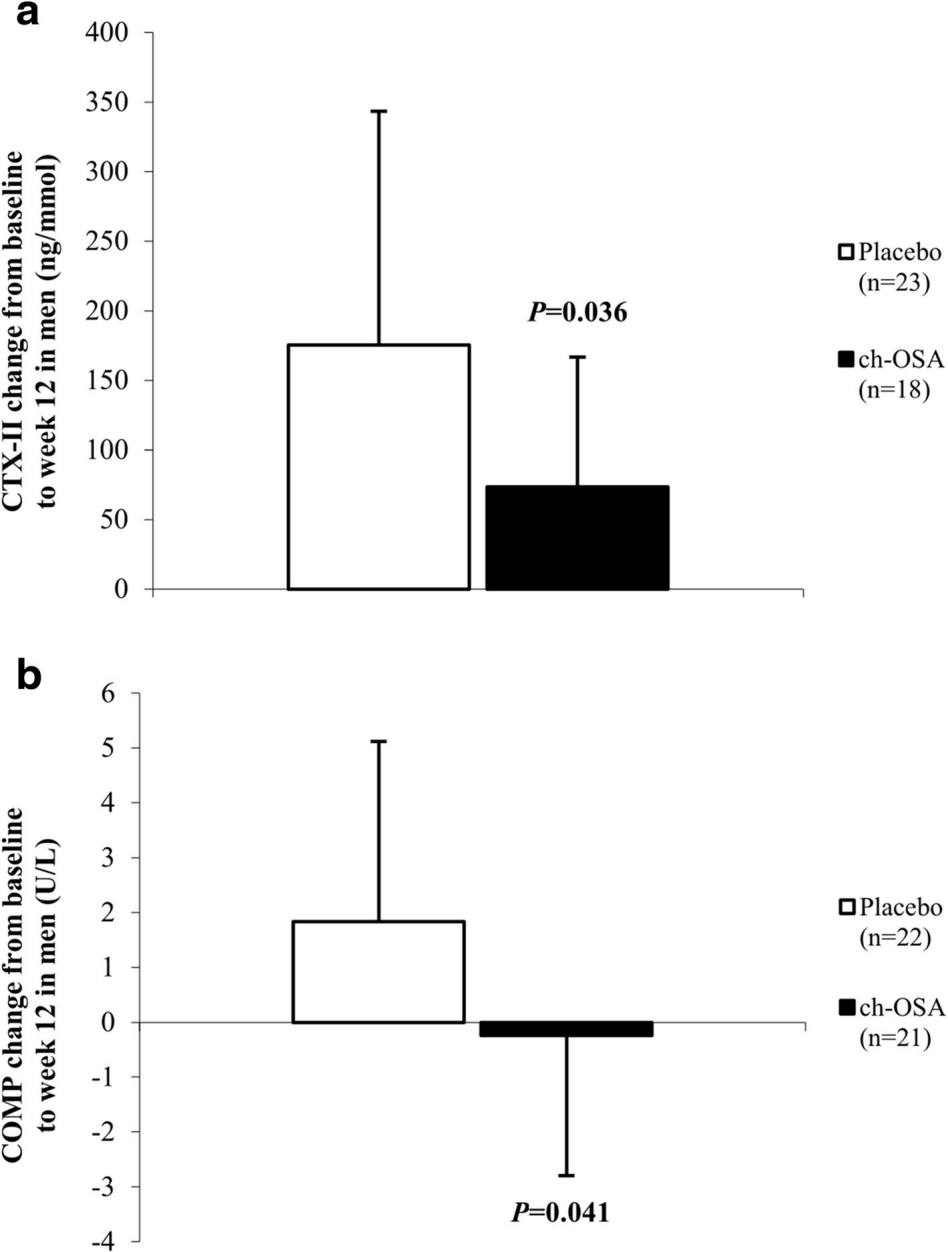
Mean change (± SD) from baseline to week 12 in CTX-II (**a**) and COMP (**b**) levels in men. *P* values refer to differences between placebo and choline-stabilized orthosilicic acid (ch-OSA)

Subsequently, pre-specified subgroup analyses by gender were performed. Table 3 provides an overview of the assessed parameters in men and women in the placebo and ch-OSA group, respectively. There was a superior effect of ch-OSA in men after 12 weeks of treatment as the mean changes from baseline in WOMAC total, WOMAC stiffness and WOMAC physical function were significantly higher with ch-OSA compared to placebo (Fig. 2). The corresponding mean differences (95% CI) between placebo and ch-OSA in men were the following: WOMAC total 11.0 mm (0.1 to 21.9), WOMAC stiffness 16.5 mm (3.0 to 30.0) and WOMAC physical function 10.6 mm (−0.5 to 21.6). A similar, yet non-significant trend was observed for WOMAC pain and Subject Global Assessment: 10.2 mm (−1.7 to 22.1) and 8.4 mm (−5.5 to 22.3), respectively. As shown in Fig. 3, increases in biomarkers of cartilage degradation (CTX-II and COMP) were significantly lower after 12 weeks in the male ch-OSA group, resulting in the following mean differences (95% CI) between placebo and ch-OSA: CTX-II 102.0 ng/mmol (12.6 to 191.4) (Fig. 3a) and COMP 2.1 U/L (0.3 to 3.9) (Fig. 3b). The statistical analysis of the female subgroups, on the other hand, demonstrated no significant treatment differences (data not shown). Similar as in the total study population, the change from baseline in silicon serum levels was significantly higher in the ch-OSA group compared to the placebo group for both women and men. The following observed mean differences (95% CI) between placebo and ch-OSA were observed: men, 26.1 μg/L (3.15 to 49.4) and women, 46.0 μg/L (27.0 to 65.0). No significant correlations were found between clinical parameters and serum silicon in neither women nor men (data not shown).

**Table 3:** Gender-specific subgroups: Overview of the absolute clinical outcome scores and biomarker values at baseline, week 2, week 6 and week 12 in the placebo and choline-stabilized orthosilicic acid (ch-OSA) group

	Placebo (*n* = 79)								ch-OSA (*n* = 87)							
	Male (*n* = 24)				Female (*n* = 55)				Male (*n* = 22)				Female (*n* = 65)			
	Baseline (mean ± SD)	Week 2 (mean ± SD)	Week 6 (mean ± SD)	Week 12 (mean ± SD)	Baseline (mean ± SD)	Week 2 (mean ± SD)	Week 6 (mean ± SD)	Week 12 (mean ± SD)	Baseline (mean ± SD)	Week 2 (mean ± SD)	Week 6 (mean ± SD)	Week 12 (mean ± SD)	Baseline (mean ± SD)	Week 2 (mean ± SD)	Week 6 (mean ± SD)	Week 12 (mean ± SD)
WOMAC total score (/100 mm)	42.0 ± 22.4	37.5^a^ ± 26.2	34.1^a^ ± 24.3	34.7 ± 30.7	43.6 ± 19.6	39.8 ± 19.0	32.1^a, b^ ± 19.9	31.2 ^a^ ± 20.5	42.5 ± 20.9	36.1^a^ ± 22.1	26.8^a^ ± 22.8	24.2^a^ ± 21.3	40.4 ± 19.0	37.7 ± 20.7	30.4^a, b^ ± 21.2	31.1^a^ ± 23.4
WOMAC pain score (/100 mm)	42.5 ± 22.7	35.1^a^ ± 24.5	32.6^a^ ± 24.0	33.0 ± 31.2	40.6 ± 19.4	37.2 ± 19.7	29.3^a, b^ ± 19.8	28.2 ^a^ ± 20.1	41.3 ± 19.3	32.2^a^ ± 20.0	24.3^a^ ± 21.5	21.6^a^ ± 19.6	37.1 ± 20.0	35.5 ± 21.9	28.8^a, b^ ± 22.0	27.6^a^ ± 22.9
WOMAC stiffness score (/100 mm)	43.8 ± 25.3	40.0 ± 27.3	33.5 ± 26.4	38.2 ± 29.6	45.9 ± 24.5	39.5^a^ ± 23.2	32.4^a, b^ ± 22.2	32.2 ^a^ ± 22.8	46.2 ± 21.8	37.2^a^ ± 25.3	28.8^a^ ± 23.8	24.1^a^ ± 23.4	43.3 ± 24.1	39.4 ± 22.5	31.1^a, b^ ± 22.6	32.0^a^ ± 25.3
WOMAC function score (/100 mm)	41.7 ± 23.3	37.9 ± 27.3	34.6 ± 25.2	34.9 ± 31.0	45.9 ± 24.5	39.5 ± 23.2	32.4^a, b^ ± 22.2	32.2 ^a^ ± 22.8	42.4 ± 22.2	27.1 ± 22.9	27.3^a^ ± 23.5	25.0^a^ ± 22.3	43.3 ± 24.1	39.4 ± 22.5	31.1^a, b^ ± 22.6	32.0^a^ ± 25.3
Subject Global Assessment (/100 mm)	51.1 ± 26.0	41.7 ± 22.4	33.3 ^a^ ± 23.4	33.8^a^ ± 24.3	48.6 ± 19.9	37.9^a^ ± 18.8	33.5^a, b^ ± 19.7	32.0 ^a^ ± 22.2	51.5 ± 18.3	36.6^a^ ± 20.9	24.6^a, b^ ± 20.8	25.8 ^a^ ± 23.7	49.6 ± 18.3	42.7^a^ ± 22.8	36.5^a, b^ ± 24.9	34.2^a^ ± 24.6
CTX-II/Creatinine (ng/mmol)	389.8 ± 178.0	-	-	565.2^a^ ± 267.8	425.6 ± 196.5	-	-	508.1^a^ ± 259.5	364.0 ± 144.7	-	-	437.4^a^ ± 171.8	454.3 ± 229.0	-	-	568.3^a^ ± 282.6
COMP (U/L)	11.1 ± 3.0	-	-	13.0^a^ ± 3.6	10.6 ± 2.7	-	-	10.6 ± 3.2	10.9 ± 2.7	-	-	10.6 ± 2.7	10.6 ± 2.9	-	-	11.3^a^ ± 2.8
Silicon (μg/L)	54.4 ± 28.1	-	-	59.2 ± 36.0	49.5 ± 35.6			67.8 ^a^ ± 44.4	68.4 ± 38.0	-	-	99.3^a, c^ ± 42.7	57.0 ± 35.3	-	-	121.3^a, c^ ± 59.1

^a^significant difference from baseline within the group; ^b^ significant difference from week 2 within the group; ^c^ significant difference between groups (placebo versus ch-OSA); -: not applicable; Number of patients without outliers for CTX-II: placebo males (*n* = 23), placebo females (*n* = 43), ch-OSA males (*n* = 18), ch-OSA females (*n* = 50); Number of patients without outliers for COMP: placebo males (*n* = 22), placebo females (*n* = 52), ch-OSA males (*n* = 21), ch-OSA females (*n* = 59); Number of patients without outliers for Silicon: placebo males (*n* = 19), placebo females (*n* = 50), ch-OSA males (*n* = 19), ch-OSA females (*n* = 56)

## Discussion

This is the first clinical study to evaluate the symptom relief effect of ch-OSA in patients with knee OA. Although the intake of the dietary supplement for 12 weeks failed to show a statistically significant benefit over placebo regarding the clinical outcome scores and biomarker values in the per-protocol population, pre-specified subgroup analyses demonstrated a significant effect of ch-OSA in men. Accordingly, WOMAC total, stiffness and physical function scores were significantly reduced in men taking ch-OSA compared to men in the placebo group. While levels of cartilage degradation related biomarkers increased over 12 weeks in men in the placebo group and in women in both groups, these levels were significantly lower in the male ch-OSA group at the final visit. More specifically, the increase in CTX-II was less pronounced and COMP levels remained unchanged. The former suggests that ch-OSA can slow the ongoing progression of cartilage degradation in men.

The management of knee OA remains a major challenge. The key objective is to cease or delay disease progression by controlling cartilage degradation, inflammation and changes of subchondral bone. Nevertheless, most published recommendations primarily aim to control OA symptoms since safe and effective disease-modifying OA drugs (DMOADs) are currently lacking [23–26]. NSAIDs, for instance, are still among the most commonly used medicines to relieve OA pain, regardless of their well-known side-effects in prolonged therapy [24, 27]. The rationale for studying the effectiveness of ch-OSA in knee OA was consequently based on its previously demonstrated effect on collagen synthesis and its safety in long-term use [14, 15]. The components of ch-OSA, choline and orthosilicic acid (OSA), have both been mentioned with respect to collagen metabolism. Firstly, choline lowers plasma homocysteine levels through its precursor-function in the biochemical conversion of homocysteine to methionine [16]. This reduction positively affects collagen cross-linking, since homocysteine has been shown to interfere with post-translational modifications of collagen through direct and indirect inhibition of lysyl oxidase as well as through down regulation of other genes involved in collagen cross-linking [28]. Indeed, Zhang et al. recently reported elevated serum homocysteine levels in patients with severe OA compared to those with mild OA or healthy controls [29]. Secondly, although the exact role of silicon in bone and connective tissue health remains unclear, its involvement in collagen synthesis and/or stabilisation was recently put forward by Jugdaohsingh et al. [30]. In the present study, the lack of correlation between the change in serum silicon and clinical parameters in men taking ch-OSA, suggests that the effect of ch-OSA cannot be explained as an effect of silicon only. ch-OSA was previously shown to have a positive effect on markers of bone formation, with a significant increase of procollagen type I N-terminal propeptide (PINP) in osteopenic women following a supplementation period of 12 months [14]. In an animal model for postmenopausal osteoporosis, it was demonstrated that ch-OSA partially prevented femoral bone loss [31].

The former supports the hypothesis of a possible effect of ch-OSA on collagen in both cartilage and subchondral bone and consequently on knee OA, particularly in view of increasing evidence of a close interrelationship between subchondral bone and articular cartilage [32, 33]. A positive effect of ch-OSA was indeed confirmed in the present study, already after 12 weeks of treatment, yet only in men. Several hypotheses can be suggested with respect to this gender difference. In general, women report more pain and disability from knee OA [34, 35]. The perception of pain, however, has been demonstrated to depend on the estrogen state. In fact, a low estrogen state was found to be related to hyperalgesia [36]. Consequently, the possibility that the enhanced pain perception masked the effect of ch-OSA in women in the present study cannot be ruled out given the predominance of postmenopausal women (98%) with low endogenous estrogen levels, of which only 7% was on hormone replacement therapy (HRT), a contraceptive, a selective estrogen receptor modulator (SERM) or phytoestrogens. In fact, the mean serum estradiol level of women in the present study was significantly lower compared to that of the male study population.

Besides the fact that postmenopausal women have a different pain perception than man, it has also been confirmed that the severity of knee OA is higher in women as well, and principally in postmenopausal women [9, 37]. Accordingly, urinary CTX-II levels were found to be significantly higher in postmenopausal women than in men [38]. The latter is in correspondence with the trend towards higher baseline CTX-II concentrations observed in – predominantly postmenopausal – women in the present study. Again, decreased estrogen levels might be of importance. Several in vitro, in vivo, genetic and clinical studies indeed designate a potential protective role for estrogens against the development of OA, given the various actions of estrogen on articular tissues [39]. Additionally, considerable experimental models indicate that estrogen deficiency-related increases in bone turnover contribute to the progression of OA [40, 41]. Therefore, in view of the more severe OA forms in women, it can be suggested that the 12-week supplementation period could have been insufficient to result in a clinical benefit.

Given the apparent extensive involvement of estrogen deficiency in the development and progression of knee OA, the use of HRTs or SERMs in women seems plausible. Nevertheless, it has been concluded that the health risks of HRTs may outweigh the potential benefits and that HRTs and SERMs should not be recommended as first-line treatments for OA [42, 43]. Several other pharmacological therapies have been studied with respect to knee OA, including symptomatic slow-acting drugs for OA (SYSADOAs) like glucosamine sulphate, chondroitin sulphate, hyaluronic acid and diacerein. Due to the frequently inconsistent conclusions of these studies, the appropriateness of the former therapies has been recently designated as uncertain [26]. Furthermore, therapeutic approaches that target subchondral bone resorption and/or formation have gained attention [32, 43]. Bisphosphonates, strontium ranelate and calcitonin have been studied in this context, yet none of these treatments has been approved as a DMOAD since evidence of concomitant structural and symptomatic effects is still pending [6, 26]. Additionally, safety issues are of importance, such as the risk of cardiovascular events associated with strontium ranelate [44]. The safety of ch-OSA in prolonged therapy, on the other hand, has been previously demonstrated [45].

Although the relevance of biochemical markers in OA has not yet been proven to be of definite clinical importance, Lotz et al. described their value as secondary outcomes [12]. More specifically, effects on biomarkers can support the primary outcome and provide evidence of pharmacodynamics and mechanisms of action of OA drugs. This is indeed valid in the present study, given that the trend observed for the primary outcome (WOMAC pain) in ch-OSA-treated men was associated with significantly lower biomarker levels, the latter referring to decreased cartilage degradation.

The present study has a number of limitations. Firstly, the evaluation of the magnitude of pain changes is ambiguous. This is related to the relatively high placebo effect – which is however common in OA trials – on the one hand, and to the complicated interpretation of changes evaluated as means rather than individual improvements, on the other hand [46]. Furthermore, the dropout rate was higher than assumed in the sample size calculation (18% in contrast to the postulated 10%), which negatively affected the statistical power in the subgroup analyses, particularly in the male subgroups.

In conclusion, this randomized, placebo-controlled trial provided a first indication of a potential benefit of ch-OSA in the treatment of knee OA in men, particularly in view of the need for a safe long-term treatment. Future research is however needed to further elucidate the observed effects and gender difference since no benefit was found in women. Considering the hypothesis that the 12-week supplementation period may have been insufficient in women, an extension of the treatment duration to at least 6 months would be valuable [47]. Additionally, the inclusion of younger, premenopausal women could, at least in part, contribute to the clarification of the role of estrogen in OA. The potential disease-modifying effects of ch-OSA at the level of the knee, on the other hand, could be evaluated after a prolonged treatment period using reliable and sensitive imaging technologies, such as MRI or high-resolution peripheral quantitative computed tomography (HRpQCT) [43, 48]. Determination of biochemical markers of bone metabolism could also extend the knowledge on the mechanisms of action of ch-OSA. Finally, application of the former research suggestions would also contribute to the identification of different subgroups. These subgroups could then be subjected to separate statistical analyses, which could provide evidence for the further assessment of ch-OSA’s potentials in view of individualized targeted treatments for knee OA.

## Conclusions

Choline-stabilized orthosilicic acid did not improve symptoms of knee osteoarthritis in the total study population but a gender effect was observed. A symptomatic improvement was found in men but not in women, after 12 weeks of supplementation, which was associated with a slight but significant reduction of biomarkers which are related to cartilage degradation. In view of the need for a safe long-term therapy for the treatment of knee osteoarthritis, this study provided first evidence on the potential benefit of choline-stabilized orthosilicic acid. Future research is however needed to further elucidate the observed effects and gender difference. Considering the hypothesis that the 12-week supplementation period may have been insufficient in women, an extension of the treatment duration to at least 6 months would be valuable.

## Additional files

Additional file 1: Table S1. Inclusion and exclusion criteria. (DOC 29 kb)
Additional file 2: Table S2. References applies to determine the relative standard deviation (power calculation). (DOCX 13 kb)
Additional file 3: Table S3. Protocol violations resulting in exclusion from the per-protocol population. (DOC 24 kb)
Additional file 4: Dataset Geusens et al. knee OA study. (XLS 112 kb)

## Abbreviations

ANCOVA: Univariate analysis of covariance
BIPED: Burden of disease, investigative, prognostic, efficacy of intervention and diagnostics
ch-OSA: choline-stabilized orthosilicic acid
COMP: Cartilage oligomeric matrix protein
CTX-II: C-terminal telopeptide of collagen type II
DMOADs: Disease-modifying osteoarthritis drugs
ELISA: Enzyme-linked immuno sorbent assay
HRpQCT: High-resolution peripheral quantitative computed tomography
HRT: Hormone replacement therapy
MRI: Magnetic resonance imaging
NSAIDs: Non-steroidal anti-inflammatory drugs
OA: Osteoarthritis
OSA: Orthosilicic acid
PINP: Procollagen type I N-terminal propeptide
RM: Repeated measures
SERM: Selective estrogen receptor modulator
SYSADOAs: Symptomatic slow-acting drugs for osteoarthritis
VAS: Visual analogue scale
WOMAC: Western Ontario and McMaster University osteoarthritis Index
