# Supplementary material for: A 12-week randomized, double-blind, placebo-controlled multicenter study of choline-stabilized orthosilicic acid in patients with symptomatic knee osteoarthritis
Source: BMC Musculoskelet Disord. 2017 Jan 5;18:2. doi: 10.1186/s12891-016-1370-7 (PMC5217239; doi:10.1186/s12891-016-1370-7)
Supplement: Additional file 1: Table S1. — Inclusion and exclusion criteria. (DOC 29 kb) [file 12891_2016_1370_MOESM1_ESM.doc]

Additional file 1: **Table S1**. Inclusion and exclusion criteria

Inclusion criteria

Between 50 and 75 years of age

Women should be either postmenopausal, surgically sterile or use an approved form of birth control

Diagnosis of primary knee OA according to the American College of Rheumatology criteria for at least 12 weeks prior to randomization i.e. knee pain plus at least three of the following characteristics:

> 50 years

< 30 minutes of morning stiffness

crepitus on active motion

bony tenderness

bony enlargement

no palpable warmth of the synovium

Radiographic confirmed Kellgren and Lawrence grade II or III (mild to moderate osteophytes and joint space narrowing) in the previous 6 months

A knee OA pain intensity score, assessed by the question “How would you describe your maximum OA knee pain when not taking analgesic medications in the 24 hour prior to this visit”, of “moderate (2)” or “moderately severe (3)” on a 5-point Likert Scale

A Western Ontario and McMaster University OA Index (WOMAC) functional subscale score > 0 on a 100 mm horizontal visual analogue scale (VAS)

Knee pain due to OA during load or movement exceeds pain from other joints, or pain experienced due to other non-articular medical conditions

Consent to continue normal physical activities throughout the study

Provision of written informed consent to participate in the study before initiating any study-related activities

Subject exclusion criteria

Unable to understand the study procedures and/or not wishing to participate in one of the subsequent therapeutic intervention protocols

Poor general health interfering with compliance or assessment

Subjects unlikely to cooperate fully in the study

Participation in another clinical trial in the previous 90 days

Pregnancy and breastfeeding

Secondary OA of the target knee, including Paget’s disease of bone, articular fracture, major dysplasias or congenital abnormalities, ochronosis, acromegaly, haemachromatosis, Wilson’s disease and primary osteochondromatosis

Morning stiffness for ≥ 30 minutes

A swollen or warm joint suspected to be secondary to gout, pseudo gout or sepsis

Significant injury in the target joint within 6 months before the start of the trial

Disease of the spine or lower extremity joints of sufficient degree to affect assessment of the target joint

Recent or current alcohol or drug abuse

Smoking of more than 28 cigarettes per day

Arthroplasty and joint surgery of the target knee within 2 years prior to the start of the study

Chondrocyte transplants in any lower extremity joint

Subjects in a high risk group for HIV

Clinically significant medical abnormalities which would make the subject unsuitable for the study as judged by the investigator

Renal failure, documented history of stroke, myocardial infarct or cancer

Treatment-related exclusion criteria: the use of medications within the specified periods prior to randomization

Topical or systemic treatment with hyaluronic acid, glucosamine sulphate, glucosamine HCl, n-acetyl glucosamine or their derivates such as chondroitin sulphate and glycosaminoglycans within 28 days

Treatment with a slow acting drug for symptom relief within 3 months

Topical and/or systemic treatment with non-steroidal anti-inflammatory drugs (NSAIDs) or analgesics that differ from paracetamol within 14 days

Use of medications with matrix metalloproteinase-inhibitory properties (e.g. tetracyclines or structurally related compounds) or oral glucocorticoids within 28 days

Use of agents claiming to possess disease or structure modifying properties (e.g. diacerhein) within 28 days

Intra-articular injection in the target knee of glucocorticoids or any other injection within 3 and 6 months, respectively

Use of food supplements containing horsetail extract, bamboo extract, silicic acid or silanol derivates within 3 months
