# Supplementary material for: A 12-week randomized, double-blind, placebo-controlled multicenter study of choline-stabilized orthosilicic acid in patients with symptomatic knee osteoarthritis
Source: BMC Musculoskelet Disord. 2017 Jan 5;18:2. doi: 10.1186/s12891-016-1370-7 (PMC5217239; doi:10.1186/s12891-016-1370-7)
Supplement: Additional file 2: Table S2. — References applies to determine the relative standard deviation (power calculation). (DOCX 13 kb) [file 12891_2016_1370_MOESM2_ESM.docx]

Additional file 2: **Table S2**. References applied to determine the relative standard deviation (power calculation)

1. Bellamy N, Bell MJ, Goldsmith CH, Pericak D, Walker V, Raynauld JP, et al. Evaluation of WOMAC 20, 50, 70 response criteria in patients treated with hylan G-F 20 for knee osteoarthritis. Ann Rheum Dis 2005;64:881-5.

2. Boureau F, Schneid H, Zeghari N, Wall R, Bourgeois P. The IPSO study: ibuprofen, paracetamol study in osteoarthritis. A randomised comparative clinical study comparing the efficacy and safety of ibuprofen and paracetamol analgesic treatment of osteoarthritis of the knee or hip. Ann Rheum Dis 2004;63:1028-34.

3. Bruyere O, Pavelka K, Rovati LC, Deroisy R, Olejarova M, Gatterova J, et al. Glucosamine sulfate reduces osteoarthritis progression in postmenopausal women with knee osteoarthritis: evidence from two 3-year studies. Menopause 2004;11:138-43.

4. Clegg DO, Reda DJ, Harris CL, Klein MA, O'Dell JR, Hooper MM, et al. Glucosamine, chondroitin sulfate, and the two in combination for painful knee osteoarthritis. N Engl J Med 2006;354:795-808.

5. Faucher M, Poiraudeau S, Lefevre-Colau MM, Rannou F, Fermanian J, Revel M. Algo-functional assessment of knee osteoarthritis: comparison of the test-retest reliability and construct validity of the WOMAC and Lequesne indexes. Osteoarthritis Cartilage 2002;10:602-10.

6. Herrero-Beaumont G, Ivorra JA, Del Carmen Trabado M, Blanco FJ, Benito P, Martin-Mola E, et al. Glucosamine sulfate in the treatment of knee osteoarthritis symptoms: a randomized, double-blind, placebo-controlled study using acetaminophen as a side comparator. Arthritis Rheum 2007;56:555-67.

7. Kim LS, Axelrod LJ, Howard P, Buratovich N, Waters RF. Efficacy of methylsulfonylmethane (MSM) in osteoarthritis pain of the knee: a pilot clinical trial. Osteoarthritis Cartilage 2006;14:286-94.

8. McAlindon T, Formica M, LaValley M, Lehmer M, Kabbara K. Effectiveness of glucosamine for symptoms of knee osteoarthritis: results from an internet-based randomized double-blind controlled trial. Am J Med 2004;117:643-9.

9. Pavelka K, Gatterova J, Olejarova M, Machacek S, Giacovelli G, Rovati LC. Glucosamine sulfate use and delay of progression of knee osteoarthritis: a 3-year, randomized, placebo-controlled, double-blind study. Arch Intern Med 2002;162:2113-23.

10. Reginster JY, Deroisy R, Rovati LC, Lee RL, Lejeune E, Bruyere O, et al. Long-term effects of glucosamine sulphate on osteoarthritis progression: a randomised, placebo-controlled clinical trial. Lancet 2001;357:251-6.

11. Theiler R, Bruhlmann P. Overall tolerability and analgesic activity of intra-articular sodium hyaluronate in the treatment of knee osteoarthritis. Curr Med Res Opin 2005;21:1727-33.

12. Tubach F, Ravaud P, Baron G, Falissard B, Logeart I, Bellamy N, et al. Evaluation of clinically relevant changes in patient reported outcomes in knee and hip osteoarthritis: the minimal clinically important improvement. Ann Rheum Dis 2005;64:29-33.

13. Zenk JL, Helmer TR, Kuskowski MA. The effects of milk protein concentrate on the symptoms of osteoarthritis in adults: an exploratory, randomized, double-blind, placebo-controlled trial. Current Therapeutic Research 2002;63:430-42.
