# Supplementary material for: A 12-week randomized, double-blind, placebo-controlled multicenter study of choline-stabilized orthosilicic acid in patients with symptomatic knee osteoarthritis
Source: BMC Musculoskelet Disord. 2017 Jan 5;18:2. doi: 10.1186/s12891-016-1370-7 (PMC5217239; doi:10.1186/s12891-016-1370-7)
Supplement: Additional file 3: Table S3. — Protocol violations resulting in exclusion from the per-protocol population. (DOC 24 kb) [file 12891_2016_1370_MOESM3_ESM.doc]

Additional file 3: **Table S3**. Protocol violations resulting in exclusion from the per-protocol population

The primary diagnosis was not valid

The OA pain intensity score of the target knee on the 5-point Likert Scale at baseline was not “moderate (2)” or “moderately severe (3)” after withdrawal of analgesic/anti-inflammatory medications

The baseline WOMAC functional score was 0

The baseline WOMAC pain subscale score is missing

A wash-out period was not respected prior to the baseline visit

A wash-out period of 2 days for paracetamol was not respected prior to each visit

Use of concomitant medication as specified in supplementary table S1 during the trial

Broken randomization code

The subject had joint surgery or arthrosopy

The subject received the wrong study dietary supplement

Compliance < 75%

Medical reason
